# Reversed scleral tunnel technique for repair of iridodialysis after blunt force trauma: a retrospective clinical study

**DOI:** 10.1186/s12886-022-02394-y

**Published:** 2022-04-15

**Authors:** Zushun Lin, Zongming Song, Zhenquan Zhao, Zhisheng Ke

**Affiliations:** 1 Eye Hospital and School of Ophthalmology and Optometry, Wenzhou Medical University, 270 Xueyuan Road, Wenzhou, Zhejiang, 325027 China; 2 Henan Provincial People’s Hospital, Henan Eye Hospital, Henan Eye Institute, 7 Weiwu Road, Zhengzhou, Henan 450003 China

**Keywords:** Iridodialysis, Reversed scleral tunnel, Closed-chamber technique, Cataract, Trauma

## Abstract

**Background:**

To investigate the efficacy and safety of reversed scleral tunnel technique for repairing iridodialysis after blunt force trauma.

**Methods:**

A total of 51 eyes of 51 patients with iridodialysis undergoing surgery were included in this study. Patients were divided into 2 groups: group A (the reversed scleral tunnel technique) and group B (the control group). Before the procedure and at 1, 3, and 6 months afterward, data on the patients’ age, gender, treatments, diagnosis, mechanism of injury, best-corrected visual acuity (BCVA), intraocular pressure (IOP), degree of iridodialysis, lens status, concomitant ocular damage, number of sutures, complications, and follow-up time were collected and compared between the 2 groups.

**Results:**

Iridodialysis was repaired and the pupil shape was restored to nearly round in all eyes. Standard phacoemulsification or lens removal was performed in all eyes. A final BCVA ≥20/60 was achieved in 13 eyes (48.1%) in Group A and 13 eyes (54.2%) in Group B. The IOP remained stable during the follow-up period in all eyes except 2 eyes (7.4%) in Group A and 3 eyes (12.5%) in Group B with angle recession. There were no statistically significant differences in BCVA and IOP between group A and group B. Intraoperatively, A significantly lower percentage of extensive subconjunctival hemorrhage occurred in Group A compared to Group B (1 eye versus 24 eyes, χ2 = 47.1, P = 0.00). Hyphema was observed in 2 eyes (7.4%) in Group A and 1 eye (4.2%) in Group B. Postoperatively, two eyes (7.4%) in Group A and 2 eyes (8.3%) in Group B developed retinal detachment. No other complications were noted during the follow-up period.

**Conclusions:**

The reversed scleral tunnel technique is a safe and effective approach for repairing iridodialysis after blunt force trauma with few complications, favorable cosmetic and visual outcomes.

**Supplementary Information:**

The online version contains supplementary material available at 10.1186/s12886-022-02394-y.

## Background

Iridodialysis is an avulsion of the iris from its natural attachment to the ciliary body at the iris root, the weakest part of the iris. It usually occurs secondary to penetrating or blunt ocular trauma or complicated intraocular surgery [1, 2]. If iridodialysis is limited to a small area or covered by the upper eyelid, patients may be asymptomatic and surgical intervention may be unnecessary. However, large iridodialysis that leads to cosmetic and functional problems, such as complaints of glare, monocular diplopia, or photophobia, often requires treatment.

Multiple techniques have been introduced for the repair of iridodialysis including open and closed chamber iridoplasty [3–11]. These methods were usually initiated with a conjunctival incision or a peritomy and required the closure of the conjunctival and scleral flaps at the end of the procedure. This impaired the native integrity of the conjunctiva and could result in conjunctival fibrosis and possible failure of trabeculectomy for glaucoma in eyes post-trauma [12]. The reversed scleral tunnel technique was first performed for scleral fixation of intraocular lenses with numerous advantages by Hoffman et al. [13]. Here, we describe a reversed scleral tunnel technique combined with closed chamber iridoplasty to address these problems and report the outcome.

## Methods

A retrospective study was performed in the Eye Hospital of Wenzhou Medical University. The study was approved by the Ethics Committee of the Eye Hospital of Wenzhou Medical University and followed the tenets of the Declaration of Helsinki. All participants signed informed consent forms. A total of 51 patients with iridodialysis secondary to blunt ocular trauma who underwent the iridodialysis repair procedure between February 2015 and January 2021 met our criterion and were included in this retrospective study. Inclusion criteria were as follows: (1) The iridodialysis was secondary to blunt trauma; (2) The degree of iridodialysis was more than 2 o’clock; (3) Postoperative follow-up period was more than 6 months; (4) Preoperative examination and follow-up data are complete.

All patients underwent a complete ocular examination before and after surgery (1, 3, and 6 months). The following data were collected from the patient’s medical records: age, gender, treatments, diagnosis, mechanism of injury, best-corrected visual acuity (BCVA), intraocular pressure (IOP), degree of iridodialysis, lens status, concomitant ocular damage such as vitreous hemorrhage, retinal detachment, number of sutures, complications, and follow-up time. The BCVA was monocularly assessed and converted to the logMAR scale with the following rules: count fingers (CF): 1.8, hand motion (HM): 2.3 logMAR units [14]. The degree of iridodialysis was assessed by slit-lamp examination. IOP was assessed and recorded as the average of three measurements.

**Fig. 1:**
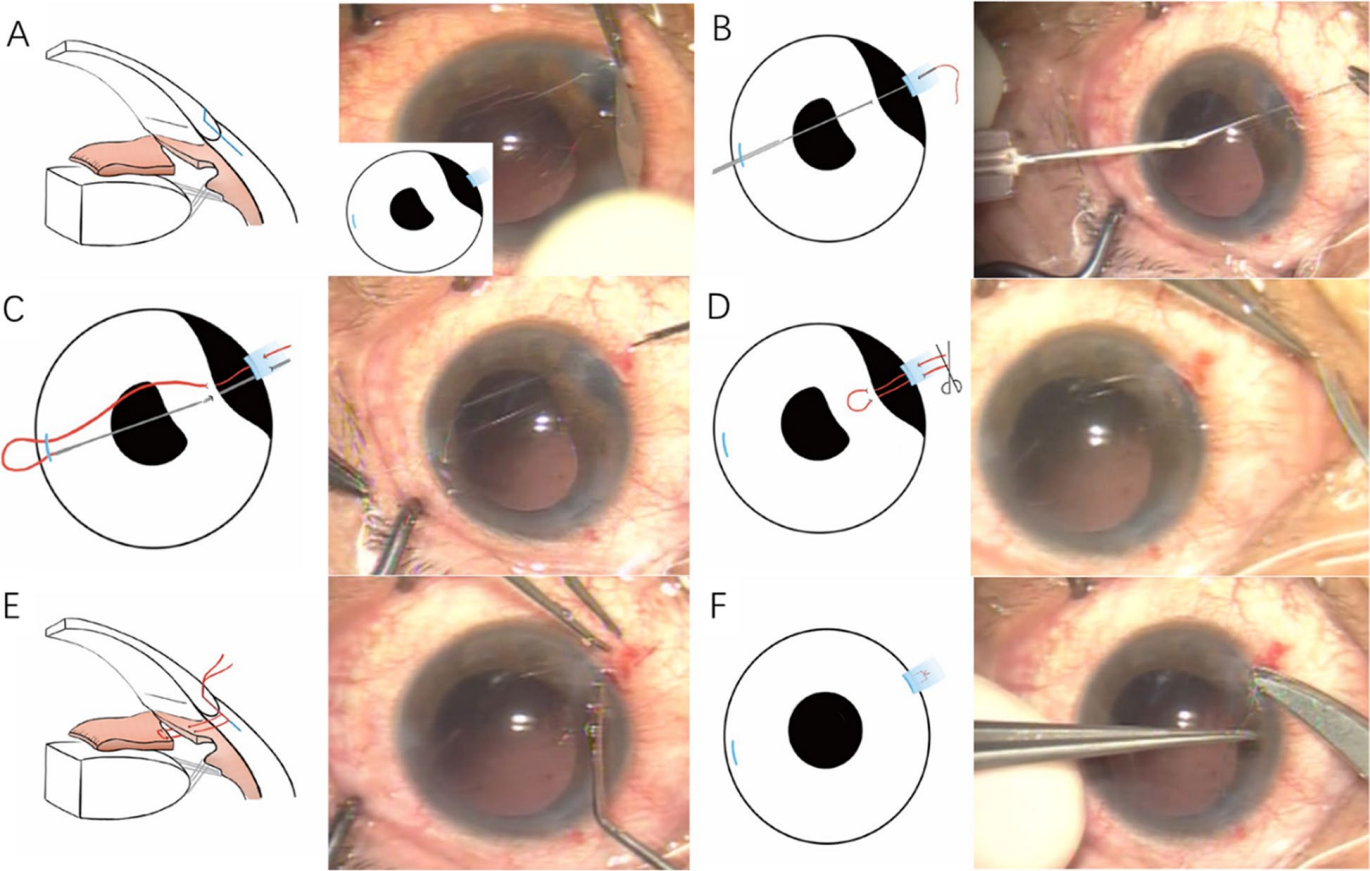
The reversed scleral tunnel technique for repair of iridodialysis. **A** Performing an incision just anterior to the limbus and creating a reversed scleral tunnel by posterior dissection along the plane of the incision. **B** A straight needle is introduced into the anterior chamber through the conjunctiva and the scleral tunnel to penetrate the iris tissue 0.5 mm from the iris base. **C** The straight needle is introduced into the anterior chamber again to penetrate a second point of the iris base and the sclera using a 26-gauge needle. **D** The needle is cut from the 10–0 polypropylene suture. **E** The suture ends are retrieved from the reversed scleral tunnel. **F** The sutures are adjusted and tied appropriately. The knot is slid into the reversed scleral tunnel

The patients were divided into Group A (the reversed scleral tunnel technique) and Group B (the control group) according to different repair procedures. In Group A, a corneal incision was made just anterior to the limbus, close to the site of iridodialysis. The incision was about 3 mm in width and approximately half of the corneal thickness in depth. A reversed scleral tunnel was created by posterior dissection along the plane of the incision. The tunnel was extended approximately 2.0 mm posteriorly from the limbus (Fig. 1A). A limbal paracentesis was performed opposite to the iridodialysis. The ophthalmic viscoelastic agent was injected to maintain the anterior chamber and stretch the iris tissue. A straight needle with a 10–0 polypropylene suture was used to penetrate the anterior chamber through the conjunctiva and the scleral tunnel 1 mm posterior to the limbus (Fig. 1B). After penetrating the iris tissue 0.5 mm from the iris base, the needle was retrieved through the limbal paracentesis. It was then re-introduced into the anterior chamber via the same paracentesis. The needle was then driven through a second point of the iris base and the sclera using a 26-gauge needle (Fig. 1 C). After that, the needle was cut from the 10–0 polypropylene suture (Fig. 1D) and the suture ends were retrieved through the external incision of the reversed scleral tunnel assisted by an allocation hook (Fig. 1E). The sutures were then adjusted and tied carefully to appropriately place the iris base and avoid covering the angle. The knot was slid into the scleral tunnel so it lay flat beneath the roof of the scleral tunnel (Fig. 1F). There was no need to close the external incision. If the iridodialysis was repaired successfully, then the procedure was complete. In cases of large iridodialysis, the same manipulations were repeated until the iris was placed at a satisfactory position. The technique is demonstrated in the additional movie file (see Additional file 1). In Group B, the iridodialysis was repaired with a double-armed suture technique [3]. A conjunctival flap was performed in the usual manner. A limbal paracentesis was performed opposite to the iridodialysis. The first needle of the double-armed suture was introduced into the anterior chamber passed through the iris base and exited out through the sclera. The second needle was introduced into the anterior chamber by the same entry site and driven through a second point of the iris base and the sclera. The suture was tied and the knot was buried. The same manipulations were repeated until the iris was restored.

All statistical analyses were performed with SPSS software v23.0 (IBM; Armonk, NY, USA). Descriptive results are shown as frequencies for categorical data and as mean ± SD for continuous variables. The independent-samples t-test was used to compare the means of continuous variables. The paired sample t-test was used for paired variables while the chi-square test was used for categorical variables. A *P* value of <0.05 was considered statistically significant.

## Results

This study included 51 eyes of 51 patients with a mean age of 55.7 ± 11.5 years. All the injuries were caused by contusion. Twenty-seven patients were assigned to group A, while 24 patients were assigned to group B. The mean time interval between injury and surgery was 39.0 days (range: 1 to 360 days) in group A and 125.2 days (range: 1 to 720 days) in group B. The mean follow-up period was 8.9 ± 5.7 months in group A and 9.2 ± 5.5 months in group B (Table 1). The characteristics of the patients were not significantly different between Group A and Group B.

**Table 1:** Characteristics of patients in this report

Characteristics	Group A	Group B	*P*-value
Patients	27	24	N/A
Age/years	58.4 ± 9.3	53.4 ± 13.6	0.187
Male (Female)	24 (3)	22 (2)	0.739
Interval between injury and surgery/days	39.0 (1–360)	125.2 (1–720)	0.06
Follow-up period/months	8.9 ± 5.7	9.2 ± 5.5	0.702
Range of iridodialysis	n/%		0.58
2–3 o’clock	13/48.1%	15/62.5%	
3–5 o’clock	9/33.3%	6/25.0%	
5–7 o’clock	5/18.5%	3/12.5%	
Number of sutures	n/%		0.73
1	13/48.1%	14/58.3%	
2	9/33.3%	7/29.2%	
3	5/18.5%	3/12.5%	

All surgeries were performed under retrobulbar anesthesia. Concomitant ocular traumatic diseases were also treated. The iridodialysis was repaired and the pupil shape was restored to nearly round in all eyes. According to the degree of iridodialysis, the iridodialysis was repaired with a single suture in 13 eyes in group A and 14 eyes in group B, with two sutures in 9 eyes in group A and 7 eyes in group B, and three sutures in 5 eyes in group A and 3 eyes in group B (Table 1).

Traumatic cataracts were observed in all cases and traumatic lens dislocation was present in 42 eyes (82.3%) before surgery. Seven eyes (13.7%) had vitreous hemorrhage, four eyes (7.8%) had retinal detachment, four eyes (7.8%) had a macular hole, nine eyes (17.6%) had retinal contusion, and five eyes (9.8%) had hyphema. Intraoperatively, retinal breaks were observed in 13 eyes (25.5%).

Standard phacoemulsification or lens removal was performed in all eyes. Vitrectomy was performed in 26 eyes (50.9%) and anterior vitrectomy was performed in 18 eyes (35.3%). A intraocular lens was placed in the capsular bag or sulcus in 32 eyes (62.8%). A three-piece intraocular lens was placed in the sulcus in the remaining 19 eyes (37.3%) during the second surgery 2 or 3 months later. The relevant parameters were not significantly different between group A and group B (Table 2).

**Table 2:** Characteristics of the surgery in this report

Characteristics	Group A	Group B	*P*-value	Total
Associated pathology				N/%
Traumatic lens dislocation	25	17	0.06	42/82.3%
Vitreous hemorrhage	4	3	0.85	7/13.7%
Retinal detachment	2	2	0.91	4/7.8%
Macular hole	2	2	0.91	4/7.8%
Retinal contusion	4	5	0.72	9/17.6%
Hyphema	4	1	0.35	5/9.8%
Retinal breaks	7	6	0.94	13/25.5%
Surgery				
Vitrectomy	13	13	0.67	26/50.9%
Anterior vitrectomy	10	8	0.78	18/35.3%
Complications				
Extensive subconjunctival hemorrhage	1	24	<0.05	25/49.0%
Hyphema	2	1	0.62	3/5.9%
Retinal detachment	2	2	0.91	4/7.8%

The mean BCVA was improved at the final follow-up visit compared with preoperatively in Group A (logMAR, 0.47 ± 0.27 versus 1.37 ± 0.60, *p* < 0.05) and Group B (logMAR, 0.51 ± 0.41 versus 1.32 ± 0.67, *p* < 0.05). A final BCVA ≥20/60 was achieved in 13 eyes (48.1%) in Group A and 13 eyes (54.2%) in Group B. The mean IOP before and after surgery was not significantly different between Group A and Group B. The IOP remained stable during the follow-up period in all eyes except 2 eyes (7.4%) in Group A and 3 eyes (12.5%) in Group B with angle recession that was controlled with antiglaucoma medication. There were no statistically significant differences in BCVA and IOP between group A and group B.

Intraoperatively, A significantly lower percentage of extensive subconjunctival hemorrhage occurred in Group A compared to Group B (1 eye versus 24 eyes, χ^2^ = 47.1, *P* = 0.00). Hyphema was observed in 2 eyes (7.4%) in Group A and 1 eye (4.2%) in Group B and eventually stabilized at the end of the surgical procedure. Postoperatively, 2 eyes (7.4%) in Group A and 2 eyes (8.3%) in Group B developed retinal detachment, which was managed by vitreous surgery. No other complications were noted during the follow-up period.

## Discussion

Numerous techniques are available to repair iridodialysis including open and closed chamber iridoplasty. Open chamber techniques including the incarceration technique described by Paton [4] and the technique using a curved needle described by McCannel [5] may increase the risk of postoperative infection. However, closed chamber techniques including Cobbler’s Technique [6], stroke and dock technique [7] and double-armed suture technique had been proven to be safer and less invasive [3]. Wan W et al. showed both the technique using a 26-gauge hypodermic needle and the technique using a double-armed polypropylene suture was effective with few complications [8]. In the present study, the reverse scleral tunnel technique was proved safe and effective compared to the double-armed suture technique.

The reversed scleral tunnel technique first described by Hoffman et al. is a common method for scleral fixation of intraocular lenses [13, 15, 16], in which the scleral tunnel is dissected posteriorly from the corneal incision. The surgeon can maintain the integrity of the conjunctiva and avoid various surgical procedures such as cauterization of bleeding, preparation of scleral flaps, suturing scleral or conjunctival flaps, thus decreasing the total surgical time and minimizing surgical risks and complications (e.g., conjunctival fibrosis, scleral thinning and knot erosion).

Our study showed that the reversed scleral tunnel technique could be an ideal companion for closed chamber iridoplasty with numerous advantages. In our study, the reversed scleral tunnel technique significantly reduces the likelihood of extensive subconjunctival hemorrhage by maintaining the integrity of the conjunctiva, and this reduced the patient’s anxiety about the cosmetic problem. There are also fewer complaints of foreign body sensation after using this technique because no sutures are left on the surface of the conjunctiva. Moreover, the knots were not rotated into the eye, thus eliminating any chance of breaking the suture and creating corectopia [9]. Meanwhile, the tightness of the iris could be easily adjusted to restore the shape of the pupil.

Regarding the visual outcomes, all eyes showed improvement in BCVA during the postoperative follow-up period compared with preoperatively. A final BCVA ≥20/60 was achieved in nearly half of the eyes in group A and group B. The postoperative BCVA was comparable with that of previous studies [10, 11]. Using a twofold technique, Narang et al. reported a final BCVA of 20/40 or better, which is similar to our results [10]. However, the improvement in visual function was not solely the result of successful iridodialysis repair. The effective treatment of cataracts and lens subluxation, as well as the maintenance of the structure and function of the retina, plays a key role in the improvement of visual function.

Hyphema occurred when penetrating the iris tissue and eventually stabilized with the help of viscoelastics in 2 eyes (7.4%) in Group A and 1 eye (4.2%) in Group B. The incidence of hyphema was comparable with that of a previous study [10]. Postoperatively, 2 eyes (7.4%) in Group A and 3 eyes (12.5%) in Group B developed elevated IOP due to angle recession and were controlled with antiglaucoma medication; this was similar to the outcomes of the study performed by Wan W et al. [8]. No other potential intraoperative or postoperative complications related to this technique, including damage to the lens capsule, angle-closure, corectopia, or infection, were observed.

The main limitations of our study are the small sample size, lack of subjective evaluation, and relatively short follow-up period. Further prospective studies, including those using a large control group, subjective evaluation and long-term follow-up are needed to confirm the results of the technique.

## Conclusion

This study revealed that the reversed scleral tunnel technique is a safe and effective approach for repairing iridodialysis after blunt force trauma with few complications, favorable cosmetic and visual outcomes.

## Supplementary Information

**Additional file 1.** Reversed scleral tunnel technique for repair of iridodialysis.

## Data Availability

The datasets used and/or analyzed in the current study are available from the corresponding author on reasonable request.

## Abbreviations

BCVA: Best-corrected visual acuity
IOP: Intraocular pressure
CF: Count fingers
HM: Hand motion
